# Supplementary material for: Diamond preservation in the lithospheric mantle recorded by olivine in kimberlites
Source: Nat Commun. 2023 Nov 2;14:6999. doi: 10.1038/s41467-023-42888-x (PMC10622582; doi:10.1038/s41467-023-42888-x)
Supplement: Supplementary file 1 — Supplementary Information [file 41467_2023_42888_MOESM1_ESM.pdf]

Supplementary Figure 1

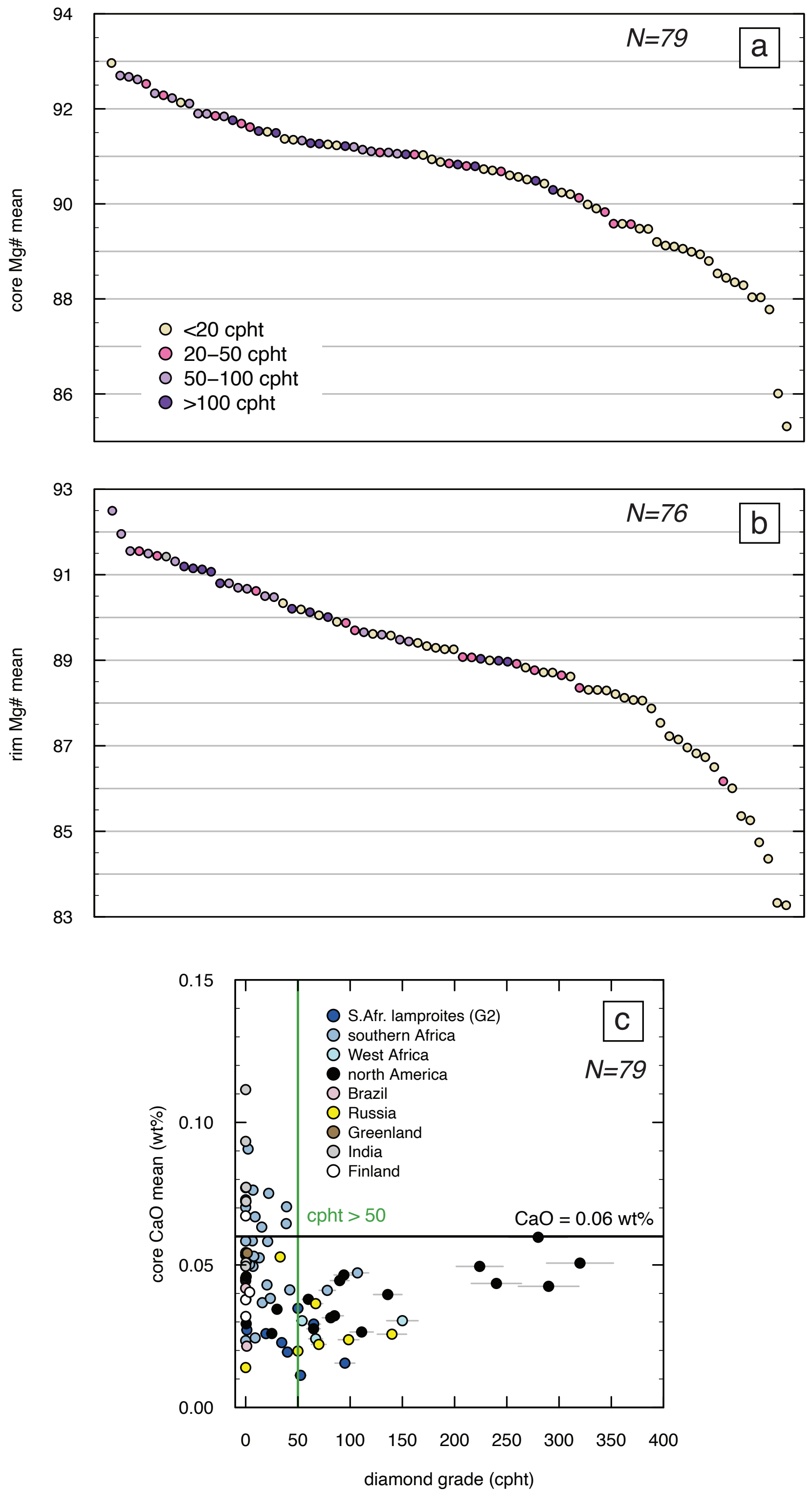

Supplementary Figure 1. Relationship between olivine composition and diamond grade in kimberlites worldwide. Olivine lamproites from South Africa and India are also included. Plots of kimberlites sorted by mean Mg# (= Mg/(Mg/Fe) as atomic proportions) of **(a)** olivine core and **(b)** olivine rim, colour-coded based on diamond grade. **(c)** Diamond grade (carats per hundred tonnes or cpht) vs mean CaO of olivine cores. The vertical light green line indicates the boundary between high and moderate to low-grade kimberlites, which is set at 50 cpht. The horizontal dark green lines, which corresponds to CaO of 0.06 wt.%, indicate the upper limit of kimberlites with high diamond grades (≥50 cpht). The error bars represent 10% of the reported value for diamond grades. (**c**) Mean Mg# vs CaO content for olivine cores colour-coded based on diamond grade showing that high diamond grades are limited to kimberlites in the upper left quadrant (Mg# ≥90.3 and CaO ≤0.06 wt.%).

Supplementary Figure 2

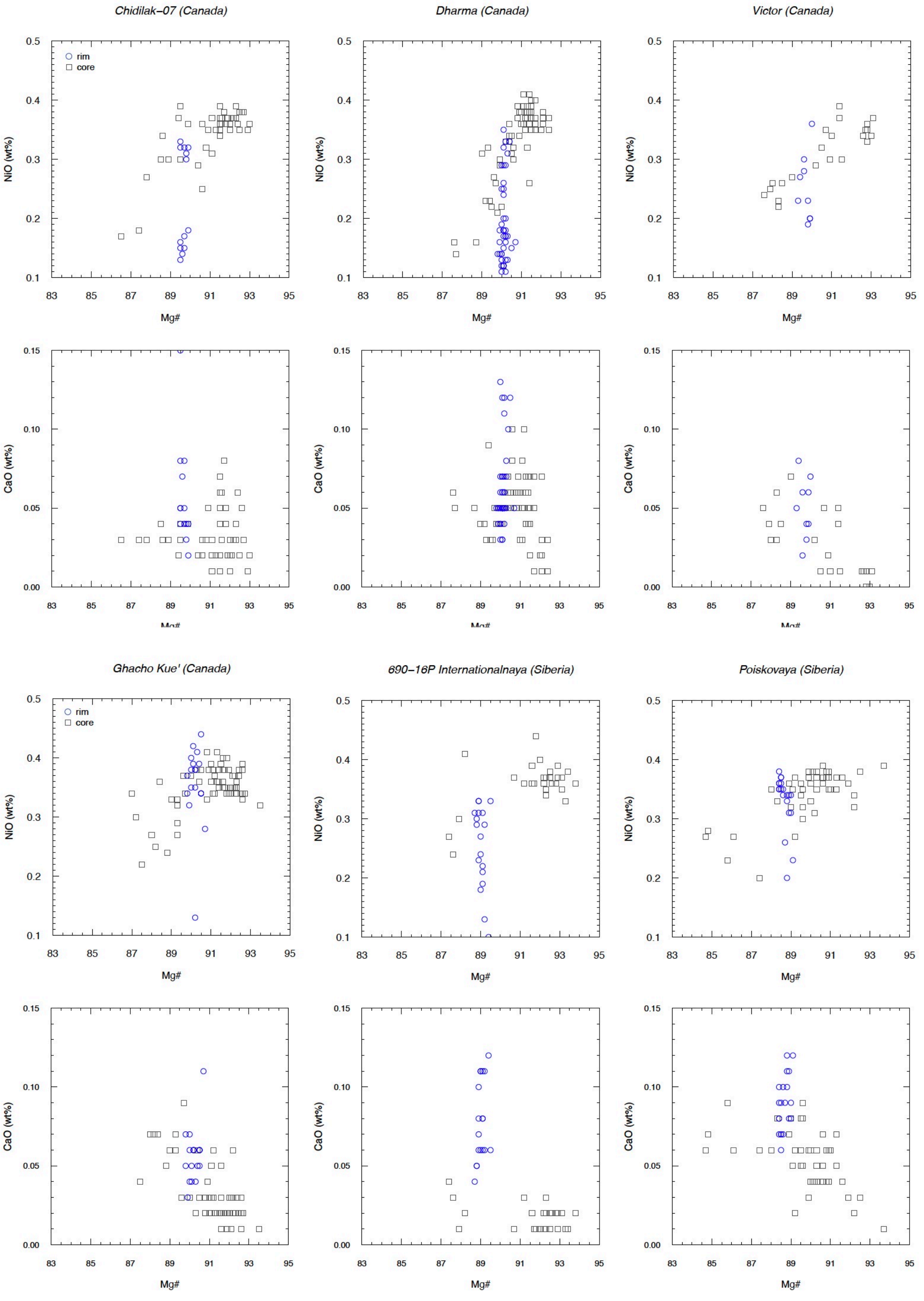

Supplementary Figure 2. New core and rim olivine compositional data for kimberlites in this study. For each locality Mg# (= Mg/(Mg/Fe) as atomic proportions) is compared to NiO and CaO concentrations.

Supplementary Figure 2, continue

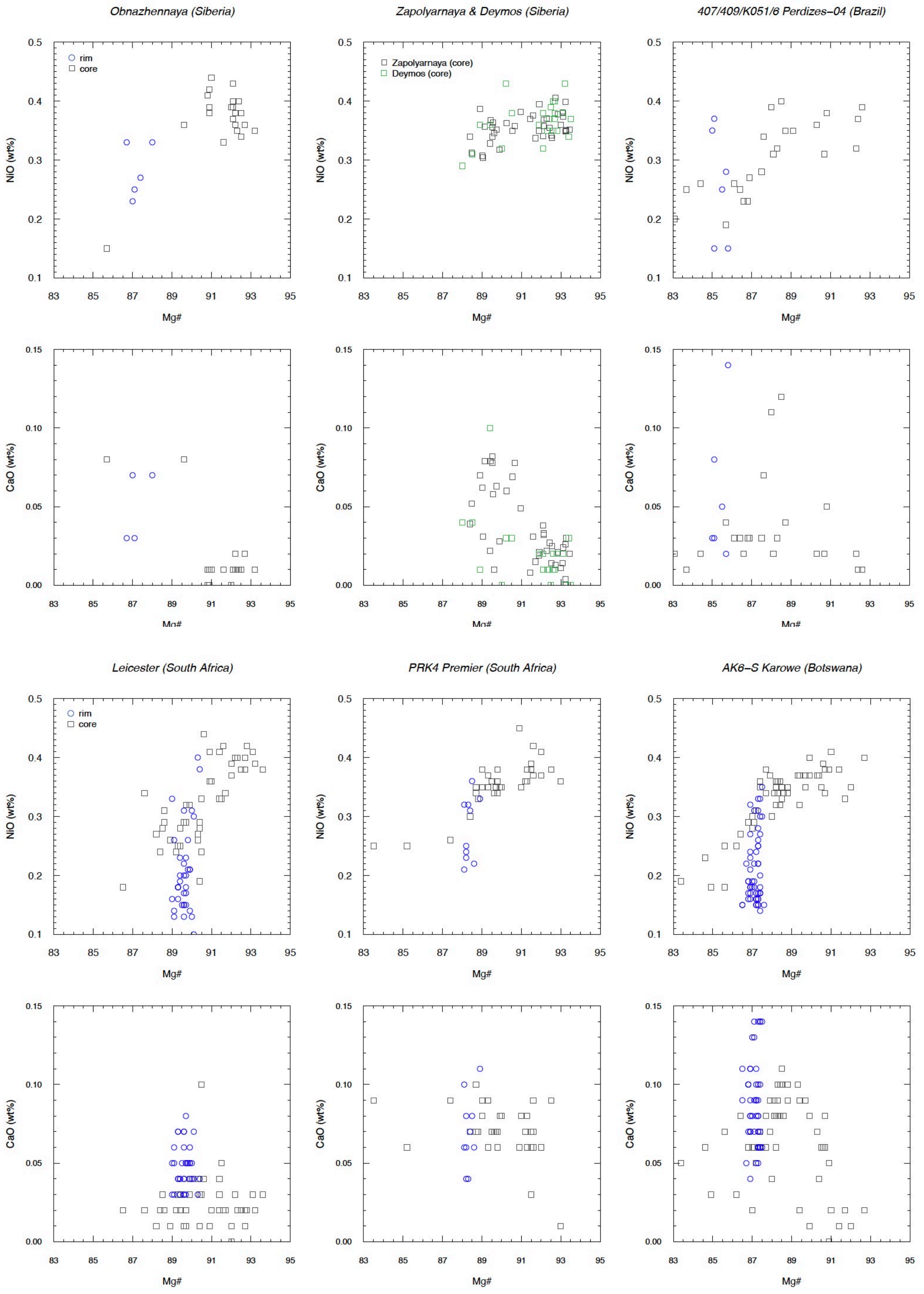

Supplementary Figure 2. New core and rim olivine compositional data for kimberlites in this study. For each locality Mg# (= Mg/(Mg/Fe) as atomic proportions) is compared to NiO and CaO concentrations.
