# Supplementary material for: Diamond preservation in the lithospheric mantle recorded by olivine in kimberlites
Source: Nat Commun. 2023 Nov 2;14:6999. doi: 10.1038/s41467-023-42888-x (PMC10622582; doi:10.1038/s41467-023-42888-x)
Supplement: Supplementary file 3 — Description of Additional Supplementary Files [file 41467_2023_42888_MOESM3_ESM.pdf]

Description of Additional Supplementary Files

Supplementary Data 1: Summary of olivine compositions, diamond grades and underlying lithospheric thickness data for kimberlites and selected olivine lamproites globally.

Supplementary Data 2: New olivine major and minor element compositions
